# Persistent Relapsing Immune Thrombocytopenia Following COVID-19 Infection

**DOI:** 10.7759/cureus.27133

**Published:** 2022-07-22

**Authors:** Blake A Boehm, Clifford D Packer

**Affiliations:** 1 Medicine, Case Western Reserve University School of Medicine, Cleveland, USA; 2 Internal Medicine, Case Western Reserve University School of Medicine, Cleveland, USA

**Keywords:** persistent itp, immune thrombocytopenia purpura, long covid, covid associated thrombocytopenia, coronavirus disease 2019 (covid-19), covid, covid 19, immune thrombocytopenia (itp), refractory itp, chronic itp

## Abstract

Immune thrombocytopenia (ITP) is a rare autoimmune disease that presents along a spectrum of disease severity, ranging from asymptomatic thrombocytopenia to potentially life-threatening bleeding complications. Recent case reports and case series suggest that a COVID-19 infection can trigger secondary ITP and may be associated with higher rates of bleeding and lower nadir platelet counts compared to patients with ITP of other etiologies. Multiple ITP relapses have also been described in some COVID-19 patients. We report the case of a 30-year-old otherwise healthy woman who presented to the hospital with fatigue, easy bruising, and a platelet count of 11 x 10^3^/µL. She responded well to our initial treatment with prednisone and intravenous immunoglobulin (IVIG) but experienced a persistent disease course with nine ITP relapses (defined as platelet count <30 x 10^3^/µL) over the next 10.5 months, requiring six additional hospital admissions for acute management as well as long-term maintenance medication adjustments. It is important for clinicians to recognize ITP as a potential complication of a COVID-19 infection and to initiate early therapy to prevent serious bleeding in these patients. Further studies will be needed to understand the natural history, optimal treatment, and prognosis for patients with relapsing COVID-19-associated ITP.

## Introduction

Immune thrombocytopenia (ITP) develops as a result of IgG autoantibodies binding platelet surface antigens, which leads to the clearance of platelets by splenic macrophages [1]. ITP is a diagnosis of exclusion and may present along a spectrum ranging from asymptomatic thrombocytopenia to serious bleeding complications such as gastrointestinal or intracranial hemorrhage [2]. The disease demonstrates a female predominance in young adults but no sex predominance in patients over 60 years of age [3-5]. ITP develops as either a primary condition or secondary to an associated trigger such as an autoimmune disease, viral infection, immunodeficiency, lymphoid malignancy, or medication [1]. Several cases of ITP secondary to COVID-19 infection have been reported, most of which are limited by short follow-up periods and limited information on the disease course and treatment response in patients with persistent or relapsing ITP. We report the case of a patient with COVID-19-associated ITP who experienced nine relapses over a period of 10.5 months.

## Case presentation

A 30-year-old previously healthy woman presented to the emergency department with a two-week history of easy bruising and fatigue eight weeks after being diagnosed with COVID-19 via PCR testing. She was unvaccinated against COVID-19 and had only mild symptoms with low-grade fever, headache, chills, and loss of taste and smell at the time of diagnosis. She was diagnosed with COVID-19 nine days after her symptoms started. Past medical history was significant for a childhood episode of thrombocytopenia and neutropenia with normal bone marrow biopsy, which resolved by four years of age and likely represented a post-viral syndrome. 

Vital signs on presentation were temperature 99.1 °F (37.3 °C), heart rate 78/min, respiratory rate 18/min, and blood pressure 109/70 mm Hg. Physical examination was remarkable for multiple ecchymoses on bilateral upper and lower extremities as well as one on the anterior chest. She did not have hepatosplenomegaly or lymphadenopathy. Laboratory evaluation demonstrated anemia (hemoglobin 10.9 g/dL, hematocrit 32.2%), thrombocytopenia (platelet count 11 x 10^3^/µL), and normal white blood cell count (8,500/µL). The baseline platelet count was 222 x 10^3^/µL approximately two years earlier. Basic metabolic panel, hepatic function panel, coagulation studies (prothrombin time (PT), partial thromboplastin time (PTT), international normalized ratio (INR)), folate, and vitamin B12 levels were within normal limits. Peripheral blood smear showed one schistocyte in every three to four high-powered fields on the day of admission, but this resolved by day three. Lactate dehydrogenase was mildly elevated to 280 U/L, and D-dimer was elevated to 1249 ng/mL. Tests for hepatitis B, hepatitis C, and HIV were negative. Antinuclear antibodies (ANA) (1:640) and anti-Ro SSA were positive. Lupus anticoagulants, anti-double stranded DNA (anti-dsDNA) antibody, anticardiolipin IgM/IgG, anti-beta 2-glycoprotein IgM/IgG, anti-Jo-1 antibody, anti-centromere B antibody, anti-Scl-70 antibody, C3/C4, anti-Sm/RNP, and ADAMTS-13 were within normal limits or negative. The patient’s presentation was inconsistent with systemic lupus erythematosus (SLE), cutaneous lupus, or Sjogren’s syndrome. After ruling out other potential causes of the patient’s thrombocytopenia, a diagnosis of ITP secondary to COVID-19 infection was made. She was treated with intravenous immunoglobulin (IVIG) 1 g/kg/day for two days and prednisone 1mg/kg/day with a resolution of the thrombocytopenia after four days. 

**Figure 1:**
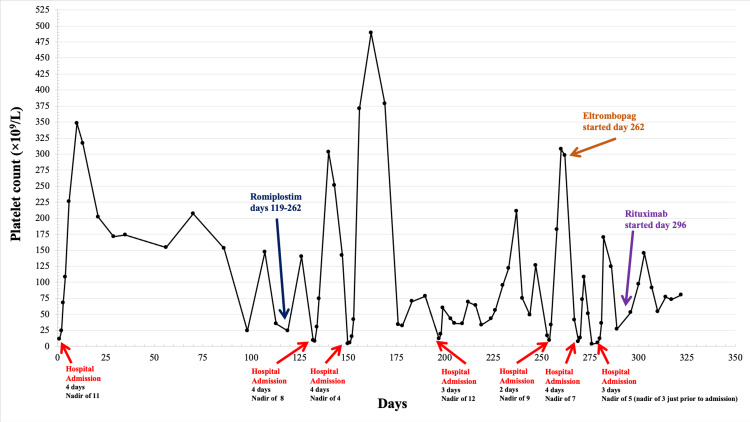
Timeline of platelet count over 10.5 months following the first thrombocytopenic event The patient was hospitalized seven times due to ITP relapses. Romiplostim was started on day 119 and discontinued on day 262, eltrombopag was started on day 262, and rituximab was started on day 296.

The patient developed a persistent disease course with nine relapses (defined as platelet count < 30 x 10^3^/µL) over 10.5 months following her initial thrombocytopenic event (Figure 1). Relapses were treated with steroids with or without intravenous Immunoglobulin (IVIG), which resolved these episodes. After two ITP relapses, the patient was started on maintenance therapy with romiplostim (thrombopoietin receptor agonist) on day 119. The patient had four relapses while on romiplostim; therefore, the medication was discontinued on day 262 due to a lack of persistent response, and she was started on eltrombopag (thrombopoietin receptor agonist). Rituximab was then started on day 296 in addition to eltrombopag. During her ITP relapses, the patient experienced mild symptoms with epistaxis, gingival bleeding, menorrhagia, fatigue, and bruising; there were no major bleeding complications.

## Discussion

Treatment for ITP typically consists of glucocorticoids, IVIG, or anti-D immunoglobulin [6,7]. Second-line therapy may need to be initiated in patients with inadequate response to primary treatment or those who experience multiple ITP relapses. Second-line treatment options primarily consist of rituximab, thrombopoietin receptor agonists (e.g., eltrombopag, romiplostim), or splenectomy [7,8]. Thrombopoietin receptor agonists are generally preferred over rituximab, while splenectomy is typically not performed until at least a year after ITP diagnosis [7]. Our patient initially responded to IVIG and prednisone; however, she continued to have relapses which required the addition of second-line therapies; thrombopoietin receptor agonists followed by rituximab. Sun et al. identified three predictors of subsequent relapse in ITP: presenting with relapsed ITP, non-O blood group, and age <25 years at the time of presentation [9]. Our patient had her initial ITP episode in early childhood, she presented with relapsed ITP after COVID-19 exposure, and her blood type was B positive. We suspect that our patient’s baseline risk factors for relapse may have predisposed her to a more severe and refractory course after COVID-19 infection.

The exact mechanism leading to thrombocytopenia in patients with COVID-19 is not yet well understood. Xu et al. have proposed multiple potential mechanisms, including bone marrow suppression, immune-mediated platelet destruction, lung injury leading to decreased platelet release from megakaryocytes in pulmonary capillary beds, and platelet consumption due to microthrombus development [10], which is a well-recognized complication of COVID-19 vasculopathy [11]. Given that our patient had an elevated D-dimer and rare schistocytes on the peripheral blood smear during her initial episode of thrombocytopenia, there may have been an early microangiopathic component to her thrombocytopenia. She then developed a prolonged disease course with recurrent ITP relapses, which may have been due to an autoimmune-mediated mechanism and/or a decrease in platelet production as a result of bone marrow infection and suppression. Chronic COVID syndrome (i.e., “long COVID”) is a well-recognized potential complication of COVID-19 infection and is believed to develop as a result of insufficient viral clearance. Chronic COVID syndrome can damage multiple organ systems, and it is possible that it may have perpetuated our patient’s ITP relapses through episodic viral attacks [12,13]. Further studies are needed to better elucidate the pathophysiology of thrombocytopenia and ITP development following COVID-19 infection and the role that chronic COVID-19 syndrome may have in the development of persistent and relapsing ITP.

Existing cases in the literature demonstrate that patients with new-onset ITP following COVID-19 tend to have higher rates of bleeding and lower nadir platelet counts compared to patients with ITP of other etiologies. However, this finding may be influenced by publication bias favoring severe ITP cases related to COVID-19 [14]. Compared to other reported cases of ITP following COVID-19, our patient was younger, had a comparable nadir platelet count at disease onset, less severe COVID-19 symptoms, and similarly adequate platelet response to IVIG and corticosteroids during the first thrombocytopenic event [14,15]. 

Table 1 compares our patient’s case with seven other reported cases of relapsing ITP following COVID-19 infection [15-18]. Our patient was significantly younger than the other seven patients, who ranged in age from 53 to 74 years. There was no gender predominance (four male, four female), and none of the patients were reported to have concurrent autoimmune diseases. The interval between the time of COVID-19 infection and initial diagnosis of thrombocytopenia ranged from zero to eight weeks [16,17], and three of the eight patients were diagnosed with ITP before they became symptomatic, presumably by routine lab testing while they were hospitalized. Our patient was not admitted to the hospital during her COVID-19 infection and was diagnosed with ITP only when she developed symptoms eight weeks later. All patients had similarly low platelet nadirs with their relapses (ranging from undetectable to 19 x 10^3^/µL, mean 7.25 x 10^3^/µL), and none experienced fatal or life-threatening bleeding complications. Our patient experienced more relapses but had a longer total follow-up time (322 days) compared to the other cases (33-100 days). Our patient had seven relapses and six hospitalizations that occurred more than 100 days after her initial episode. This suggests that a longer period of follow-up might be necessary to characterize the natural history of relapsing COVID-19-associated ITP. Our patient was not vaccinated for COVID-19; vaccination status was not reported for the other seven patients with relapsing ITP. More data on vaccination status in patients with relapsing COVID-19-associated ITP would help to determine if unvaccinated patients are at higher risk for this serious complication. 

**Table 1:** Case reports of recurrent ITP following COVID-19 infection ITP - immune thrombocytopenia; PLT - platelet; IVIG - intravenous immunoglobulin; DEX - dexamethasone; PDN - prednisone; mPDN - methylprednisolone; HTN - hypertension; HLD - hyperlipidemia; CR - complete response Complete response (CR) defined as platelet count returning to at least 100x10^9^/L Response defined as platelet count returning to between 30-100x10^9^/L and at least doubling of the baseline platelet count * ITP was diagnosed 27 days after the development of the first COVID-19 symptoms. The patient tested negative for COVID-19 using RT-PCR but clinical symptoms and chest CT were highly suggestive of COVID-19 infection ** Undetectable platelet count therefore recorded as <3x10^9^/L

Patient	Author	Gender and age (years)	Comorbid medical conditions	Initial ITP symptoms	Time from COVID-19 diagnosis to first ITP episode	Number of ITP relapses	Nadir platelet count (x10^9^/L)	Treatment	Total follow-up (days)	Outcome
1	Our case	F, 30	None	Ecchymosis, fatigue	Eight weeks	Nine	3	IVIG 1g/kg/day for two days and PDN. Relapses: PDN or DEX, with or without IVIG. Romiplostim from day 119 to 262, Eltrombopag started on day 262, rituximab started on day 296	322	Multiple relapses alternating with response or CR
2	Mahevas et al. [17]	F, 62	Not reported	No bleeding seen	Nine days	One	9	PDN for five days	60	Response then relapse on day 58
3	Mahevas et al. [17]	M, 65	Not reported	No bleeding seen	One day	One	17	DEX for four days	60	CR then relapse on day 30
4	Mahevas et al. [17]	M, 53	Not reported	Purpura	27 days *	One	19	PDN for three weeks and IVIG for three days	50	CR then relapse on day 35
5	Bennett et al. [18]	F, 73	HTN, HLD	None	Concurrent	One	< 3 **	One unit of PLT, and IVIG 1 g/kg/day for two doses. Relapse: IVIG and DEX	33	CR then relapse on day 33
6	Kewan et al. [15]	M, 63	HTN	Petechiae, mucosal bleeding, epistaxis	30 days	One	2	DEX 40 mg for four days, IVIG for two days, and eltrombopag on days 5-28	61	CR then relapse (day not specified)
7	Serrano et al. [16]	F, 60	None	Ecchymosis, petechiae, retinal hemorrhages	Four weeks	Three	2	mPDN 1 mg/kg/day for two weeks. First relapse: four doses of rituximab 375 mg/m^2^/week IV, IVIG 1g/kg/day for two days, and DEX 40 mg/day for four days. Second relapse: DEX, IVIG, four doses rituximab, and eltrombopag. Third relapse: IVIG, eltrombopag, and DEX	100	Response, relapse on day 21, CR, relapse on day 35, CR, relapse on day 49, CR
8	Serrano et al. [16]	M, 74	Severe intellectual disability	Epistaxis and melena	Eight weeks	Two	6	PLT transfusion, IVIG 1 g/kg/day for two days. First relapse: IVIG, and DEX 40 mg/day for four days. Second relapse: IVIG, and eltrombopag 50 mg/day started	100	CR, relapse day 22, CR, relapse day 57, CR

## Conclusions

ITP can lead to severe thrombocytopenia, which predisposes patients to potentially life-threatening bleeding complications. COVID-19-associated ITP generally responds to standard treatment for ITP, but in some cases, patients may suffer multiple relapses and require long-term treatment with thrombopoietin receptor agonists or rituximab. We report the case of a 30-year-old woman who had nine ITP relapses and seven hospitalizations over 10.5 months following COVID-19 infection. Additional research is needed to determine the risk factors for relapsing COVID-19-associated ITP and better characterize the natural history, prognosis, and optimal treatment for this condition. Physicians should consider the diagnosis of ITP in the setting of thrombocytopenia following COVID-19 infection and initiate early treatment to prevent serious bleeding complications.
